# Optimizing Electrode Montages of Transcranial Direct Current Stimulation for Attentional Bias Modification in Early Abstinent Methamphetamine Users

**DOI:** 10.3389/fphar.2018.00907

**Published:** 2018-08-10

**Authors:** Alireza Shahbabaie, Javad Hatami, Ali Farhoudian, Hamed Ekhtiari, Ali Khatibi, Michael A. Nitsche

**Affiliations:** ^1^Department of Psychology and Neurosciences, Leibniz Research Center for Working Environment and Human Factors, Dortmund, Germany; ^2^Institute for Cognitive Science Studies, Tehran, Iran; ^3^Iranian National Center for Addiction Studies, Tehran University of Medical Sciences, Tehran, Iran; ^4^Faculty of Psychology and Educational Sciences, University of Tehran, Tehran, Iran; ^5^Substance Abuse and Dependence Research Center, University of Social Welfare and Rehabilitation Sciences, Tehran, Iran; ^6^Department of Psychiatry, Tehran University of Medical Sciences, Tehran, Iran; ^7^Department of Psychology, Bilkent University, Ankara, Turkey; ^8^Interdisciplinary Program in Neuroscience, Bilkent University, Ankara, Turkey; ^9^Department of Neurology, University Medical Hospital Bergmannsheil, Bochum, Germany

**Keywords:** attentional bias, probe detection task, drug addiction, craving, transcranial direct current stimulation (tDCS), methamphetamine, non-invasive brain stimulation (NIBS), electrode montage

## Abstract

**Introduction:** Chronic use of most psychoactive drugs may lead to substance dependence and drug addiction. Drug addiction is a chronically relapsing disorder, and current pharmacological and behavioral therapies are not fully efficient. Attentional bias (AB) is hypothesized to have a causal contribution to substance abuse, addiction development and, maintenance. Transcranial direct current stimulation (tDCS) has been of increasing interest in the past few years as a means for modulating neuroplasticity of the human brain. Although several studies have reported promising therapeutic effects for tDCS in drug abusers, there is no consensus about optimal electrode montages and target brain regions. This study was aimed to compare effectiveness of several electrode montages in modifying AB.

**Methods and Materials:** Ninety early-abstinent methamphetamine users were recruited from several residential drug-rehabilitation centers in Tehran province. They were randomly assigned to six groups with different electrode montages, targeting the left or right dorsolateral prefrontal cortex (DLPFC) as follows: Two conditions with anodal tDCS over the right DLPFC (return electrode placed over the left shoulder or left supraorbital ridge), three conditions with the anode positioned over the left DLPFC (return electrode over the right shoulder, right supraorbital ridge, or contralateral DLPFC), and one sham condition. Active stimulation intensity was 2 mA DC, delivered for 13 min followed by a 20-min rest and another 13 min of stimulation. The probe detection task (PDT) was performed to assess AB. The positive and negative affect scale (PANAS), and the depression anxiety stress scales (DASS) were used to assess baseline affective status before the intervention.

**Results:** Mixed model analysis showed that the left DLPFC/right shoulder and left DLPFC/right DLPFC montages reduced AB toward drug-cues in comparison with sham stimulation.

**Conclusion:** Our findings indicate that anodal stimulation over the left DLPFC reduces AB in methamphetamine users. This study offers promising findings for further studies investigating tDCS as a clinical device to modify AB in drug users.

## Introduction

Addiction is a chronic and relapsing brain disease, characterized by a compulsion to seek and take the drug, and accompanied by a negative emotional state when the drug is not accessible (George and Koob, 2017). The 2017 World drug report by the United Nations Office on Drugs and Crime (UNODC) states that approximately 250 million people between the age of 15 and 64 used drugs at least once in 2015 (United Nations Office on Drugs and Crime, 2017). The widespread use of methamphetamine and disorders related to amphetamines makes this substance very relevant for the global burden of diseases attributable to drug use disorders. Amphetamines are at the second position, after opioids (United Nations Office on Drugs and Crime, 2017). Methamphetamine is a powerful and highly addictive stimulant, affecting the central nervous system. More specifically, it affects frontostriatal brain regions such as the striatum, prefrontal cortex, and cingulate cortex, causing deficits in cognitive control and selective attention, beyond other symptoms (Nordahl et al., 2003; Salo et al., 2005, 2007).

Recent studies have suggested that cognitive processes are affected by the motivational salience of drug-related cues in drug dependents (Stacy and Wiers, 2010; Manning et al., 2017). This cognitive bias affects attention resources, which are captured selectively and automatically by drug-related cues (Ernst et al., 2014). It moreover either causes or indexes processes that initiate substance-seeking behavior (Field et al., 2009). Consequently, drug-related attentional bias (AB) has been hypothesized to have causal effects on drug use, addiction development, maintenance, and relapse (Robinson and Berridge, 1993; Franken, 2003; Field, 2005; Weinstein and Cox, 2006). According to the suggested role of AB in the perpetuation of and relapse to addiction, diverse methods (e.g., pharmaceutical and cognitive-behavioral) have been applied to reduce AB (Field et al., 2014). However, only a few double-blind studies are available for effects of AB retraining on treatment outcomes or relapse prevention (Begh et al., 2013).

Neural models of drug-related attention bias suggested that AB is derived from a reciprocal relation between reward salience and cognitive control networks (Hester and Luijten, 2014). The reward salience network includes the nucleus accumbens (Nac) (Nestor et al., 2011), hippocampus and amygdala (Janes et al., 2010; Vollstädt-Klein et al., 2012), and has been associated with reinforcing properties of addictive drugs, specifically it reinforces allocation of attention resources toward drug-related stimuli. In contrast, the cognitive control network is able to decrease AB to drug-cue stimuli by dorsolateral prefrontal cortex (DLPFC), and medial prefrontal – including the rostral and dorsal anterior cingulate cortex (ACC) – activity (Hester and Garavan, 2009; Janes et al., 2010; Luijten et al., 2011, 2012; Nestor et al., 2011; Vollstädt-Klein et al., 2012). Although drug dependents typically show increased activity of the cognitive control network, this is often insufficient during exposure to drug cues, because of simultaneous activity in the reward network (Kober et al., 2010; Hester and Luijten, 2014). In line with this neural model, recent studies revealed that facilitation of DLPFC activity improves AB modification, underscoring the pivotal role of the DLPFC for modification of attention allocation to salient stimuli (Clarke et al., 2014; Heeren et al., 2015).

Non-invasive brain stimulation (NIBS) techniques provide promising opportunities for exploring the role of brain regions for cognitive processes, and for altering respective processes. Transcranial direct current stimulation (tDCS), one of the most common NIBS techniques, delivers a weak direct electrical current through scalp electrodes to the brain and induces stimulation polarity-dependent alterations of cerebral excitability and activity (Stagg and Nitsche, 2011; Woods et al., 2015). With conventional stimulation protocols, anodal stimulation enhances cortical excitability (Nitsche and Paulus, 2001), and cathodal stimulation produces an opposite effect (Nitsche et al., 2003b). The primary mechanism of action is a subthreshold neuronal membrane depolarization and hyperpolarization, respectively, which are induced by anodal and cathodal stimulation (Nitsche et al., 2009). Long-lasting effects of tDCS share some similarities with long-term potentiation and depression (LTP and LTD) of glutamatergic synapses, as suggested by pharmacological studies in both, animals (Rohan et al., 2015) and humans (Liebetanz et al., 2002; Nitsche et al., 2003a, 2004). Although the method is still experimental, preliminary findings suggest that tDCS might be a potential clinical treatment for drug addiction (Feil and Zangen, 2010; Yavari et al., 2015), such as nicotine (Fregni et al., 2008; Boggio et al., 2010; Fecteau et al., 2014; Meng et al., 2014), alcohol (Boggio et al., 2008; Nakamura-Palacios et al., 2012; da Silva et al., 2013; Klauss et al., 2014), cocaine (Conti and Nakamura-Palacios, 2014; Conti et al., 2014; Gorini et al., 2014), marijuana (Boggio et al., 2010), heroin (Wang et al., 2016), and methamphetamine dependency (Shahbabaie et al., 2014, 2018). Although these studies have reported promising therapeutic effects, electrode montages differ between studies, and it has not been sufficiently explored which montage results in optimal effects. Although these studies have reported promising therapeutic effects, stimulation protocols differ between studies, and it is unclear which protocol results in optimal effects. This is not a trivial problem, because at the cellular level, the response of an individual neuron to electrical current depends on the distance from the current source, which determines electrical field strength, neuronal orientation with regard to the electrical field as well as morphology of the neuron (Das et al., 2016). In accordance, not only the target, but also the return electrode position is relevant for stimulation effects, because it determines electrical field orientation and current flow direction in the brain. Consequently, the electrode montage is a crucial factor for the effectiveness of this technique (Bikson et al., 2010; Moliadze et al., 2010). Therefore, the main purpose of this study was to explore the efficacy of different tDCS montages on AB in drug addicts. For this purpose, we applied five different electrode montages with different return electrode positions, but identical current intensity, electrode size and stimulation duration, in order to explore the efficacy of these electrode montages on AB to drug cues. The stimulation target was the DLPFC due to its role as a hub of the attentional control network in abstinent methamphetamine users.

## Materials and Methods

### Participants

Early abstinent methamphetamine users who volunteered for attending the current study were enrolled from several residential centers with an abstinence-based program located in a rural area near Tehran. We recruited only male volunteers, because sex differences of dopamine release in the brain in response to methamphetamine consumption, as well as an impact of the menstrual cycle on the subjective effects of the drug have been reported (Sofuoglu et al., 2002; Evans and Foltin, 2006; Munro et al., 2006; Riccardi et al., 2006). Initially, 96 subjects were recruited based on the following inclusion and exclusion criteria. Inclusion criteria were: (1) right-handed, as indicated by an Edinburgh Handedness Inventory score larger than 60, (2) normal or corrected-to-normal vision, (3) a history of at least 12 months methamphetamine use before entering the center’s current program, (4) a history of methamphetamine consumption for at least 3 days a week in the last month before entering the current program at the above-mentioned center, and (5) abstinence from any sedative or stimulant drug except for nicotine, as confirmed by a negative urine test. Exclusion criteria were: (1) any major current neurological and psychiatric disorders except for substance-related disorders, (2) history of substance-induced psychosis in the last 6 months, (3) any current use of drugs affecting the central nervous system except nicotine, (4) history of traumatic brain injury, migraine, epilepsy, and tumors, (5) cranial or brain metal implant. Ninety male subjects, between the age of 20 and 45 (mean = 30.76, *SD* = 6.178), completed the whole study procedure. Participants were naïve to tDCS. They were randomly assigned to one of six groups with different electrode montages (only the tDCS technician was informed about the assignment). The study was designed and carried out according to the ethical principles of the Declaration of Helsinki, approved by the Independent Ethics Committee (IEC) of the University of Social Welfare and Rehabilitation Sciences USWR.REC.1393.160, and was registered in the Iranian Registry of Clinical Trials (IRCT) under registration number: IRCT2014031611234N2. All subjects signed a written informed consent form before participation.

### Transcranial Direct Current Stimulation

**FIGURE 1:**
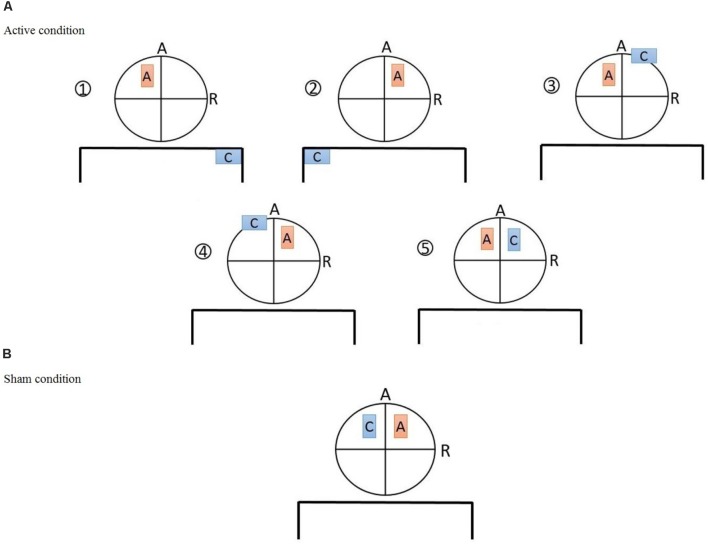
**(A)** The five active tDCS montages: (1) Unilateral monopolar (UM-L): anode positioned over F3, cathode over the right shoulder, (2) unilateral monopolar (UM-R): anode positioned over the F4 and the cathode over the left shoulder, (3) unbalanced bilateral bipolar (UBB-L): anode positioned over F3 and the cathode over the right supraorbital ridge, (4) unbalanced bilateral bipolar (UBB-R): anode positioned over F4 and the cathode over the left supraorbital ridge, (5) balanced bilateral bipolar (BBB-L): anode positioned over F3 and the cathode over F4 **(B)** sham tDCS montage: anode positioned over F4 and cathode over F3.

Direct current was delivered through a battery-driven, constant current stimulator (Eldith, NeuroConn GmbH, Germany) and was transferred by a pair of 5 cm × 7 cm (35 cm^2^) electrodes. Electrodes were standard carbonic, covered by saline-soaked sponge cases. Subjects were randomly assigned to one of the six groups with different electrode montages (**Figure 1**) targeting the left/right DLPFC (F3/F4 based on 10-20 EEG system) as follows:

(1) Unilateral monopolar left (UM-L): Anode positioned over the left DLPFC, cathode over the right shoulder
(2) Unilateral monopolar right (UM-R): Anode positioned over the right DLPFC and the cathode over the left shoulder
(3) Unbalanced bilateral bipolar left (UBB-L): Anode positioned over the left DLPFC and the cathode over the right supraorbital ridge
(4) Unbalanced bilateral bipolar right (UBB-R): Anode positioned over the right DLPFC and the cathode over the left supraorbital ridge.
(5) Balanced bilateral bipolar left (BBB-L): Anode positioned over the left DLPFC and the cathode over the right DLPFC
(6) Sham condition: one electrode positioned over the right DLPFC and the other over the left DLPFC

Active stimulation was conducted with the first five of the aforementioned montages in which 2 mA direct current was delivered for 13 min followed by a 20-min rest and another 13 min of intervention (13:20:13). This design is suggested to induce longer-lasting neurophysiological effects, as compared to a single session of tDCS (Monte-Silva et al., 2013). To ensure that participants were in a fairly homogeneous mental state during the 20 min break of the stimulation sessions, they were shown a movie featuring abstract shapes (Vanderwal et al., 2015). The same procedure was used for the sham condition except that the stimulator was turned off after ramping up and down, which took 30 s, at the start of the stimulation session.

### Pictorial Probe Detection Task (PDT)

The task was programmed and presented via MATLAB v. R2014b 8.4.0 (Mathworks, Inc., Natick, MA, United States) on a Windows 7 operating system. Participants were seated approximately 60 cm away from a 20″ LED Monitor (SAMSUNG, S20C32575B PLUS) with 1600 × 900 resolution and 60 Hz refreshing rate to perform the probe detection task (PDT) which consisted of eight practice and 172 test trials.

Every trial started with a fixation cross (500 ms), positioned at the middle of the screen. Then, two pictures (size: 5 cm × 8 cm, see section “Images” for a detailed description), with 5 cm margins from the center of the screen, appeared for 500 ms on the left and right half of the monitor. Immediately (16.67 ms) after fading of the two pictures, an arrow probe pointing up or down was presented at the location of one of the pictures (left or right). Participants were asked to press the respective button on a standard (QWERTY) keyboard as quickly and accurately as possible to indicate the probe direction. The arrow remained on the screen until the subject responded. In the congruent trials, probe and target stimuli (drug cues) appeared on the same side of the screen, while in the incongruent trials probe and target stimuli appeared on the opposite sides of the screen. Participants had to press the **Y** key with their right index finger when the probe’s direction was upward (left or right location) and the **B** key with their left index finger when the direction was downward (left or right location). Y and B keys were labeled with ↑ and ↓ respectively. The inter-trial interval (ITI) was randomized between 800 and 1,200 ms. Every picture was presented four times with an equal number of probe and picture location (left and right). The order of the direction of the arrows was counterbalanced; the stimuli were presented in two different random orders, before and after brain stimulation.

### Images

The stimuli in the PDT consisted of 90 color pictures with solid black background, which were adjusted for resolution and brightness. Thirty of the images were a series of methamphetamine-related cues including images from crystalized methamphetamine, instruments for consumption, people who are using the substance, and other items which are associated with drug consumption (Ekhtiari et al., 2010; Shahbabaie et al., 2014). These were chosen from previous studies based on scores of induced craving (Ekhtiari et al., 2010). The other 60 images were neutral. Half of them were paired in order to be used in the filler (12 paired neutral images), warmup (two paired neutral images) and buffer trials (one paired neutral image). The other 30 neutral images were created to match the methamphetamine-related cues for visual complexity, composition, brightness, and figure-ground relationships. All images were successfully pilot-tested with a healthy control group of 20 subjects to ensure that participants could identify their contents and judge whether they were drug-related.

### Questionnaires—Measures of Affective Status

#### Positive and Negative Affect Schedule

The positive affect and negative affect schedule (PANAS) is a 20-item self-report measure that assesses two distinctive dimensions, positive mood (10 items) and negative mood (10 items) (Watson et al., 1988). Participants rated each item on a five-point Likert scale, where 1 indicates not experiencing that specific affect and 5 indicates experiencing a high level of a specific mood.

#### Depression Anxiety Stress Scales

The depression anxiety stress scales (DASS) is a measure of negative affect, which is widely used in clinical and non-clinical populations of adults. The DASS-21 was developed to provide a self-report measure of anxiety, depression and tension/stress, and includes 21 statements. Participants expressed their amount of agreement with each statement on a four-point Likert scale from no agreement (0 = not at all) to a high level of agreement (3 = always).

### Procedure

**FIGURE 2:**
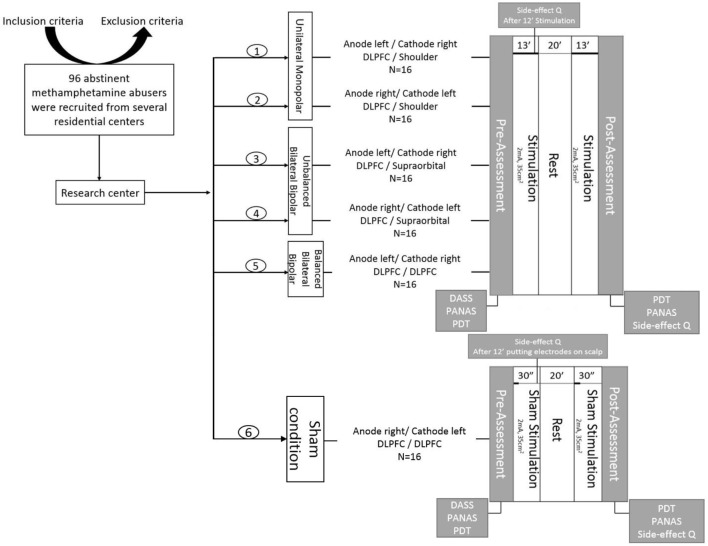
General procedure: First, volunteers were recruited based on the inclusion/exclusion criteria. Then, they were informed about the experiment and signed an informed consent form. Participants were randomly allocated to six groups, including one sham-controlled and five experimental groups with different electrode montages over the dorsolateral prefrontal cortex (DLPFC). Afterwards, the depression anxiety stress scales (DASS-21) were completed. The positive and negative affect schedule (PANAS) and the probe detection task (PDT) were performed before and after brain stimulation. Additionally, subjects were asked to fill in the side effect questionnaire after 12 min of stimulation and at the end of experiment.

This study was performed in a single per participant session based on a randomized, case-controlled, double-blinded design. First, participants were chosen due to inclusion/exclusion criteria. Second, we asked the participants to read the subject information sheet and sign the consent form, if they agreed to participate. Third, basic demographic information, including a history of drug use and addiction treatment was recorded via a structured interview conducted by an expert drug counselor. Then participants were randomly allocated to one of the six groups. Subjects were asked to complete the PANAS and the DASS-21 in order to assess their baseline affective state before conduction of the experiment. Afterwards, the subjects were seated on a comfortable chair to perform the PDT in a separate, quiet cognitive lab. The maximum time required for this task was 7 min. Then they received one of the interventions based on the group assignment in a brain stimulation lab. Afterward, they returned to the cognitive lab for post-intervention assessments, including PDT and PANAS. Eventually, we assessed any possible side effects using a tDCS side-effect checklist both during stimulation (exactly 12 min after the start of stimulation) and at the end of the intervention session (the procedure is illustrated in **Figure 2**). Participants and the evaluating investigators were blinded to the intervention type. Only the tDCS technician, who was not further involved in data acquisition and analysis, applied tDCS montages based on a prior randomized block design table.

### Statistical Analysis

Statistical analysis was performed using SPSS v. 24.0.0 (IBM Corp., Armonk, NY, United States). Initially, the demographic variables of the six groups were compared using a Kruskal–Wallis *H* test in order to confirm that the randomization was successful and the group means were identical for these parameters.

#### Preprocessing of the PDT

Initially, we explored between-group differences of error counts by a Kruskal–Wallis test. Then, outlier trials were eliminated. These were trials with RTs deviating more than ±2 SDs from the individual mean as well as RTs shorter than 200 ms and longer than 2,000 ms (Koster et al., 2004). These outliers and errors were excluded from any further analyses.

#### Processing of the PDT

At first, two paired sample *t*-test were conducted; one to compare incongruent and filler trials and another to compare congruent and filler trials at baseline assessment. Significant differences will confirm presence of bias in allocation of attentional resources to drug-related cues in early abstinent methamphetamine users before tDCS. A mixed model 3-factorial analysis of variance (ANOVA) with the dependent variable reaction time (RT), the within-subject factors time point (two levels: before and after stimulation), and trial type (three levels: congruent, incongruent, and filler), and the between-subject factor electrode montage (six levels: UM-L, UM-R, UBB-L, UBB-R, BBB-L, and BBB-R) was conducted. In case of a significant three-way interaction, to improve interpretability of the data, and to identify the direction of changes of AB, we calculated two indices of AB:

Disengagement⁢ bias⁢ index =incongruent⁢ trials−neutral⁢ trials

Engagement⁢ bias⁢ index =congruent⁢ trials−neutral⁢ trials.

These subscales allowed us to test whether tDCS modified the engagement bias of attention toward drug cues, and/or the disengagement bias of attention away from drug cues (Koster et al., 2004). Then we conducted two 2-factorial ANOVAs for both, disengagement bias and engagement bias indices, separately. Dependent on significant results of the ANOVAs, explorative *post hoc* independent samples two-tailed *Student’s t*-tests were conducted to compare each tDCS montage with sham stimulation. *Post hoc* test *p*-values were corrected for multiple comparisons based on the Šídák method. The critical level of significance was *p* < 0.05 for all statistical tests.

#### Side-Effects

We assessed the intensity of side effects related to different montages during and after stimulation. A one-factorial ANOVA with the between-subject factor electrode montage was performed for each side effect for the during and after stimulation time points. In case of significant effects in the ANOVA, independent sample *post hoc Student’s t*-tests were conducted.

## Results

First, the homogeneity of our intervention groups for demographic variables was confirmed. The Kruskal–Wallis *H* test showed no significant differences between the six groups (with different tDCS montages) with respect to age (χ^2^ = 8.448, *df* = 5, *p* = 0.133), level of education (χ^2^ = 4.382, *df* = 5, *p* = 0.496), duration of addiction (χ^2^ = 3.463, *df* = 5, *p* = 0.629), and duration of abstinence (χ^2^ = 4.268, *df* = 5, *p* = 0.511). In addition, mean scores of all subscales of the DASS-21 including depression (χ^2^ = 2.409, *df* = 5, *p* = 0.790), anxiety (χ^2^ = 4.629, *df* = 5, *p* = 0.463), and stress (χ^2^ = 1.474, *df* = 5, *p* = 0.916) were not statistically different between the six groups. Similarly, the comparison of pre-intervention PANAS subscales including positive affect (χ^2^ = 3.251, *df* = 5, *p* = 0.661) and negative affect (χ^2^ = 1.534, *df* = 5, *p* = 0.909) revealed no significant inter-group differences. Moreover, a repeated measures ANOVA confirmed the absence of an interaction between baseline RT in the different trial types and tDCS intervention groups [*F*(8.90, 149.59) = 1.31, *p* = 0.23]. Also the main effect of montages was not significant [*F*(5, 84) = 0.796, *p* = 0.556], but the main effect of trial types was strongly significant [*F*(1.78, 149.59) = 72.30, *p* < 0.0001], as expected.

### Attentional Bias (PDT)

The error rate was fairly low (*M* = 6.99, range 0–21) and not significantly different between intervention groups (*H* = 4.596, *df* = 5, *p* = 0.467). The number of outliers per participant ranged from 0 to 7 (*M* = 4.81). Errors and outliers accounted for only 3.50% of the data and were excluded from further analyses. Preprocessed mean RT of congruent, incongruent, and filler trials are shown in **Table 1**.

**Table 1:** RT data (in ms) of the probe detection task (congruent, incongruent, filler trials).

Montages	Type	RT pre-tDCS		RT post-tDCS		RT changes	
				
		*Mean*	*SD*	*Mean*	*SD*	*Mean*	*SD*
UM-L	Filler	513.34	58.32	503.13	56.49	10.21	23.97
	Incongruent	542.51	63.11	517.85	54.65	24.66	32.54
	Congruent	544.83	66.65	523.05	55.42	21.79	34.91
UM-R	Filler	500.26	50.60	487.54	58.52	12.72	15.81
	Incongruent	522.63	57.39	507.43	64.08	15.20	25.72
	Congruent	524.69	56.04	510.59	65.06	14.10	26.49
UBB-L	Filler	526.87	37.59	496.86	29.97	30.01	23.91
	Incongruent	555.48	50.91	508.48	39.10	47.00	33.80
	Congruent	551.15	47.96	523.14	41.54	28.01	26.34
UBB-R	Filler	543.91	72.73	523.89	69.97	20.01	36.45
	Incongruent	558.12	71.37	538.50	77.24	19.62	46.07
	Congruent	552.29	71.71	540.39	76.36	11.89	40.82
BBB-L	Filler	508.61	58.76	493.12	57.82	15.49	19.91
	Incongruent	530.54	61.50	503.66	62.68	26.89	30.65
	Congruent	524.81	60.05	501.97	56.90	22.84	19.82
BBB-R	Filler	523.39	44.31	505.81	54.84	17.57	21.90
	Incongruent	545.94	52.16	528.72	51.58	17.22	17.77
	Congruent	543.90	47.52	535.77	53.02	8.13	15.29


**FIGURE 3:**
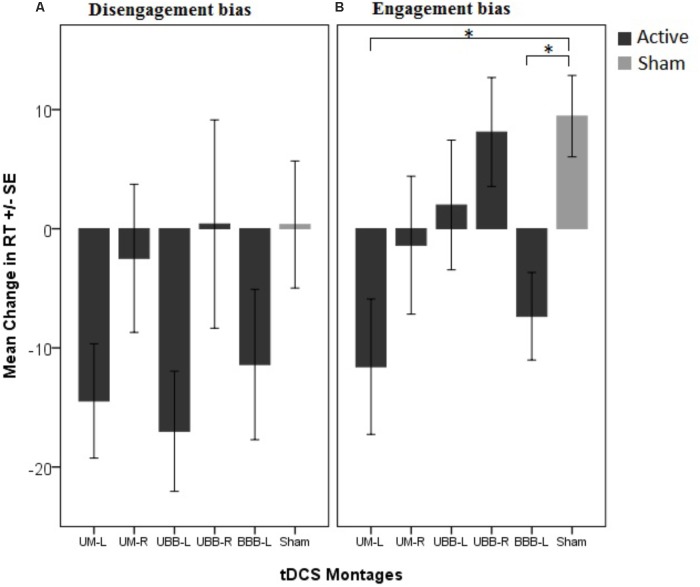
Mean change in attentional bias indices in 5 different tDCS montages in comparison with the control group (sham condition). **(A)** The disengagement bias index indicates changes in attentional bias away from drug cues induced by tDCS and **(B)** the engagement bias index indicates the change of attentional bias toward drug cues induced by tDCS. ^∗^*P* < 0.05.

An AB was observed in the baseline assessment. The paired samples *t*-tests showed a significantly longer RT in both, congruent (*df* = 89, *t* = 9.26, *p* = 0.0001, Cohen’s *d* = 0.35) and incongruent (*df* = 89, *t* = 9.38, *p* = 0.0001, Cohen’s *d* = 0.39) trials in comparison with the filler trial. The repeated measures ANOVA conducted for RTs shows that the main effect of electrode montage [*F*(5, 84) = 0.74, *p* = 0.59, $\eta_{p}^{2}$ = 0.04] was not significant, but revealed significant main effects of trial type [*F*(2, 168) = 105.34, *p* < 0.001, $\eta_{p}^{2}$ = 0.04] and time [*F*(1, 84) = 50.77, *p* < 0.001, $\eta_{p}^{2}$ = 0.38]. The two-way interactions between congruency and group [*F*(10, 168) = 1.41, *p* = 0.182, $\eta_{p}^{2}$ = 0.07] and between time points and group were not significant [*F*(5, 84) = 1.26, *p* = 0.28, $\eta_{p}^{2}$ = 0.07], while the two way interaction between congruency and time points [*F*(2, 168) = 6.98, *p* = 0.001, $\eta_{p}^{2}$ = 0.08] was significant. Importantly, the predicted three-way interaction between trial type, electrode montage and time point was also significant, [*F*(10, 168) = 1.84, *p* = 0.05, $\eta_{p}^{2}$ = 0.1]. In order to ease interpretation of this three-way interaction, we conducted two 2-factorial ANOVAs for both, disengagement bias and engagement bias indices, separately. The repeated measures ANOVA conducted for the disengagement bias index shows no significant main effect of electrode montage [*F*(5, 84) = 0.67, *p* = 0.64, $\eta_{p}^{2}$ = 0.03] while the main effect of time point was significant [*F*(1, 84) = 8.57, *p* = 0.004, $\eta_{p}^{2}$ = 0.09]. Additionally, the two-way interaction between time point and electrode montage was also not significant [*F*(5, 84) = 1.56, *p* = 0.17, $\eta_{p}^{2}$ = 0.08]. However, the 2-factorial ANOVA conducted for the engagement bias index shows a marginal significant difference for the main effect of electrode montage [*F*(5, 84) = 2.18, *p* = 0.06, $\eta_{p}^{2}$ = 0.11] while the main effect of time point was not significant [*F*(1, 84) = 0.004, *p* = 0.95, $\eta_{p}^{2}$ = 0.0001]. Importantly, the predicted two-way interaction was significant *F*(5, 84) = 2.95, *p* = 0.016, $\eta_{p}^{2}$ = 0.15]. The *post hoc* analysis with independent sample *t*-tests revealed that the engagement bias toward drug cues decreased in the UM-L (*df* = 28, *t* = -3.170, *p* = 0.019, Cohen’s *d* = 1.157) and BBB-L montages (*df* = 28, *t* = -3.345, *p* = 0.009, Cohen’s *d* = 1.221) relative to sham stimulation (**Figure 3**). Furthermore, to confirm that the results are not affected by changes in neutral conditions (filler) after tDCS, we conducted a respective one-way ANOVA. Results showed no significant differences between groups [*F*(5, 84) = 1.13, *p* = 0.35, $\eta_{p}^{2}$ = 0.63].

It is worth mentioning that none of the reported changes are attributable to changes in mood, as confirmed by analysis of the PANAS results. According to the results of the respective one-way ANOVA, mood (post-pre) in the positive [*F*(5, 89) = 0.58, *p* = 0.71] and negative[*F*(5, 89) = 1.46, *p* = 0.21] affect scores was not significantly different across different montages.

### Side-Effect Analysis

The ANOVAs conducted for the side effect questionnaires showed a significant difference between montages in intensity of tingling during stimulation, but not after stimulation. There were no significant differences for headache, vertigo, itching, dizziness, drowsiness, and nausea during or after 26 min of stimulation, as illustrated in **Table 2**. *Post hoc* independent *t*-tests showed a higher intensity of tingling during UM-L (*df* = 28, *t* = 3.365, *p* = 0.003), UBB-L (*df* = 28, *t* = 3.251, *p* = 0.003), and BBB-L (*df* = 28, *t* = 3.214, *p* = 0.003) montages compared to the sham condition. Pearson’s correlation showed, however, that the effect of tDCS on RT in the UM-L (filler *p* = 0.87, incongruent *p* = 0.42, congruent *p* = 0.71) and UBB-L (filler *p* = 0.24, incongruent *p* = 0.88, congruent *p* = 0.65) and BBB-L (filler *p* = 0.08, incongruent *p* = 0.15, congruent *p* = 0.48) montages was not significantly correlated with tingling scores during stimulation.

**Table 2:** The intensity of side effects was analyzed by one-way ANOVAs with the between subject factor electrode arrangement.

	Side effects	*df*	*F*-value	*P*-value
During stimulation	Headache	5, 84	0.581	0.714
	Vertigo	5, 84	0.933	0.464
	Tingling	5, 84	2.714	0.025^∗^
	Itching	5, 84	1.84	0.114
	Dizziness	5, 84	0.545	0.742
	Drowsiness	5, 84	0.228	0.949
	Nausea	5, 84	0.8	0.553
After stimulation	Headache	5, 84	0.974	0.438
	Vertigo	5, 84	0.465	0.801
	Tingling	5, 84	1.232	0.301
	Itching	5, 84	1.354	0.25
	Dizziness	5, 84	0.671	0.646
	Drowsiness	5, 84	0.84	0.525
	Nausea	5, 84	1	0.423


*A significant effect of tingling, but no significant effect of other side effects was revealed. UM-L, monopolar unilateral-left anode; UM-R, monopolar unilateral-right anode; UBB-L, unbalanced bipolar bilateral-left anode; UBB-R, unbalanced bipolar bilateral-right anode; BBB-L, balanced bipolar bilateral-left anode; BBB-R, balanced bipolar bilateral-right anode; RT, reaction time. ^∗^α = 0.05.*

## Discussion

The current study offers the first empirical evidence that lateralized stimulation of the DLPFC contributes to the modification of drug-related AB depending on return electrode position. In the present study, we sought to investigate the effect of tDCS over the DLPFC with different electrode montages on AB modification. Our results show that, compared to the sham condition, anodal stimulation over the left DLPFC significantly decreased the engagement bias toward drug cues in abstinent methamphetamine users when the return electrode was positioned on the right shoulder or contralateral DLPFC.

Control and experimental groups were homogeneous regarding demographic and affective state variables. Age, level of education, duration of addiction, and duration of abstinence did not differ between groups. In addition, there were no differences in depression, anxiety, and stress subscales of the DASS-21 between groups. Moreover, positive and negative affects did not show any significant inter-group differences before, and after intervention. This is in line with previous studies, suggesting modulation of emotional processing and attentional processing of emotional stimuli with NIBS over the DLPFC, without specifically influencing mood (Nitsche et al., 2012; Mondino et al., 2015).

Baseline PDT analysis demonstrated that the participants had a longer RT in both congruent and incongruent trials compared to neutral trials. As we expected, early abstinent methamphetamine users showed biases in attentional resource allocation to drug-related cues. This is in accordance with results of other studies reporting that drug users fixate longer on drug-related cues compared to neutral cues, when the probe follows either a drug-related cue or a neutral cue (e.g., Waters et al., 2003; Munafò et al., 2005; Bradley et al., 2008; Chanon et al., 2010; Field et al., 2013). However, results of other studies suggest that drug users have a tendency to approach drug- related cues faster compared to neutral cues (e.g., Bradley et al., 2004; Constantinou et al., 2010; Zhao et al., 2018). This controversy may be due to different task features in AB assessment (e.g., longer or shorter stimulus presentation), different AB formula (contrasting congruent and incongruent trials, rather than comparing them to neutral trials), drug users explored at different stages of addiction (e.g., intoxication, withdrawal, early abstinent, long-term abstinent) or different drug dependency (e.g., heroin, alcohol, nicotine methamphetamine, etc.).

The AB modification in the UM-L montage group demonstrated in the present study is consistent with a recent tDCS study using the same electrode montage (Wolkenstein and Plewnia, 2013). They showed that AB to emotional stimuli was completely abolished in people who suffer from major depression disorder via a similar stimulation protocol. Are few more studies reported a comparable modification of AB via a left DLPFC anodal/extracephalic cathodal electrode arrangement (Clarke et al., 2014; Chen et al., 2017; Heeren et al., 2017), however, in these studies the return electrode was placed over the ipsilateral shoulder, which is different from the montage used in the present study. Additionally, by showing that anodal stimulation over the left DLPFC facilitates attentional control on drug-related cues, our data confirm and extend results of previous studies, suggesting that the DLPFC is relevantly involved in top-down attention control (Ochsner and Gross, 2005; Hester and Luijten, 2014).

The significant AB modification in the BBB-L condition is consistent with a study by Brunoni et al. (2014), who report a significant modification of negative AB in depression with anodal tDCS via a similar electrode montage. These results are in line with the frontal asymmetry hypothesis, which suggests that asymmetrical frontal cortical brain activation, in most cases shown for alpha frequency bands in the EEG, enhances allocation of attentional resources to subjectively significant stimuli (Duecker and Sack, 2015; Adolph et al., 2017). In accordance, a rightward shift of frontal asymmetry is associated with a respective AB to salient cues in anxiety and depression (Mathersul et al., 2008; Miskovic and Schmidt, 2010; Grimshaw et al., 2014). Therefore, it can be speculated that the effective reduction in AB toward drug cues in the BBB-L montage is a consequence of the tDCS-induced enhanced activity of the left DLPFC in combination with reduced activity by synchronous cathodal tDCS over the right DLPFC, and thus due to a re-balancing effect. Since we, however, did not include a condition with sole right DLPFC stimulation, the contribution of this area to the effects remains to be clarified in future studies. It is important to note that all electrode arrangements with a specific effect of tDCS on AB included a left DLPFC activity enhancement. This is in accordance with numerous studies showing higher evidence for drug-related AB modification by left, as compared to right DLPFC activity enhancement for drug-induced craving (Boggio et al., 2008, 2009, 2010; da Silva et al., 2013). In further accordance, a recent meta-analysis showed that exposure to drug cues in drug dependents are associated with leftward asymmetry (Gordon, 2016). This, as well as our finding, are interpretable within the neural model suggested by Hester and Luijten (2014). They argue that the reward salience and cognitive control networks are two opposing forces involved in emergence and modification of drug-related AB (Hester and Luijten, 2014). Exposure to drug cues activates reward salience, and thus down-regulates processing in emotion- and reward-related regions (e.g., amygdala, insula, and striatum), but up-regulates control networks (e.g., lateral prefrontal and dorsal anterior cingulate) coincidently, in order to allocate attentional resources to task-relevant stimuli. However, considering the strong reward network and weakened cognitive control network in drug dependents, increased activity of the control network appears to be insufficient to overcome the activity of the reward network and thus results in continued AB to drug cues. Therefore, it might be speculated that the tDCS-induced facilitation over the left DLPFC as a proxy region for attentional control in drug addicts in this study resulted from enhanced efficacy of the respective control network.

Some limitations of this study should be taken into account. (I) Although 90 abstinent methamphetamine dependents participated in the current study, the number of participants assigned to each group (*n* = 15) was relatively low. (II) We did not explore physiological effects of stimulation in this study, which would have extended our knowledge about mechanistic aspects of electrode montages as well as AB. (III) The current study was limited to abstinent and treatment-seeking patients, who have different patterns of brain activity during cognitive task performance as compared to active drug users who do not seek treatment (Chang et al., 2006). (IV) The case-control design of the current study does not exclude the possibility that inter-individual differences had an impact on the results completely. This could be resolved by conducting a similar experiment with a crossover design, which could be the focus of future studies. (V) In this study, we only explored the impact of different electrode montages on AB. However, to optimize stimulation effects, other relevant parameters, such as current density, stimulation duration, electrode size, and configuration should be also considered in future.

Therefore, future studies with larger sample size, different stimulation intensity, and duration, electrode sizes, and shapes as well as studies exploring physiological effects and inter-individual differences are required to improve our understanding of the underlying mechanisms of tDCS effects on AB and select the most effective tDCS protocol for AB modification.

## Conclusion

To conclude, our findings show that anodal stimulation over the left DLPFC reduces AB in abstinent methamphetamine users with different return electrode positions, while anodal stimulation of the right DLPFC had no effects. This study thus offers a potentially promising way for tDCS as a rehabilitation tool for AB modification. In order to accomplish this goal, future studies are needed to extend our understanding of the physiological effects of tDCS over the DLPFC, and our model-based knowledge about the impact of current distribution, including its dependency from the return electrode position, and its relation to neuronal orientation, to optimize stimulation protocols accordingly.

## Author Contributions

AS, AK, MN, HE, and JH conceived the conception and design of the study. AS and AF contribute on data collection. AS analyzed the data in collaboration with MN and AK. AS prepared the draft of the manuscript. All authors contributed to editing the manuscript and the approved final version was submitted for publication.

## Conflict of Interest Statement

MN is member of the Scientific Advisory Board of Neuroelectrics. The remaining authors declare that the research was conducted in the absence of any commercial or financial relationships that could be construed as a potential conflict of interest.
